# Retraction: A new method for ethical and efficient evidence generation for off-label medication use in oncology (a case study in glioblastoma)

**DOI:** 10.3389/fphar.2026.2021924

**Published:** 2026-10-13

**Authors:** Frontiers Editorial Office

The journal retracts the 27 June 2019 article cited above.

Following publication, the Medicines and Healthcare products Regulatory Agency found that the METRICS study described in this article was a clinical trial conducted without appropriate ethics approval or informed consent. In addition, data were found to contain inaccuracies and lack quality control. This constitutes a breach of Frontiers’ guidelines and those of the Committee on Publication Ethics. As such, the article is being retracted.

This retraction was approved by the Chief Executive Editor of Frontiers. The authors received a communication regarding the retraction and had a chance to respond. This communication is recorded by the publisher.

